# Silencing of ultradian rhythms and metabolic depression during spontaneous daily torpor in Djungarian hamsters

**DOI:** 10.1007/s00360-024-01573-1

**Published:** 2024-07-08

**Authors:** Gerhard Heldmaier, Luzie Braulke, Johanna Flick, Thomas Ruf

**Affiliations:** 1 Animal Physiology, Department of Biology, Marburg University, Karl-von-Frisch Str. 8, 35032 Marburg, Germany; 2 Institute of Wildlife Ecology, Veterinary University, Vienna, Austria

**Keywords:** Wavelet analysis, IR thermovision, Hypometabolism, Propranolol, Mirabegron, Body temperaure

## Abstract

Ultradian rhythms of metabolism, body temperature and activity are attenuated or disappear completely during torpor in Djungarian hamsters, for all three ultradian periodicities (UR*small*, UR*medium* and UR*large*). UR*small* and UR*medium* disappear during entrance into torpor, whereas UR*large* disappear later or continue with a low amplitude. This suggests a tight functional link between torpor and the expression of ultradian rhythms, i.e. torpor is achieved by suppression of metabolic rate as well as silencing of ultradian rhythms. Spontaneous torpor is often initiated after an ultradian burst of activity and metabolic rate, beginning with a period of motionless rest and accompanied by a decrease of metabolic rate and body temperature. To extend previous findings on the potential role of the adrenergic system on torpor induction we analysed the influence of the ß3-adrenergic agonist Mirabegron on torpor in Djungarian hamsters, as compared to the influence of the ß-adrenergic antagonist Propranolol. Hamsters were implanted with 10 day release pellets of Mirabegron (0.06 mg day^−1^) or Propranolol (0.3 mg day^−1^). Mirabegron transiently supressed and accelerated ultradian rhythms but had no effect on torpor behaviour. Propranolol did not affect torpor behaviour nor the expression of ultradian rhythms with the dosage applied during this study.

## Introduction

During torpor metabolic rate is suppressed to a fraction of the euthermic resting metabolic rate and metabolic pathways are rerouted to burn lipids. Many small mammals use torpor to reduce their long term energy expenses, either as “short daily torpor” for about 2 through 16 h during the resting phase of their circadian rhythm or as multiday torpor bouts during hibernation (for review see Ruf and Geiser 2015). The regular appearance of daily torpor during the resting phase of the circadian rhythm suggests a link between the circadian clock and torpor. Occasionally torpor bouts can occur several times during 24 h (Oelkrug et al. 2011) suggesting that torpor can also be part of the ultradian organisation of behaviour.

A potential role of ultradian rhythms (URs) for the timing of torpor has been noticed before (Braulke and Heldmaier 2010; Meyer et al. 2012; Diatroptov et al. 2019). A recent analysis of URs of metabolic rate (MR) revealed the existence of three different URs, UR*small*, UR*medium* and UR*large* with ultradian periods in the range of 1 h, 1.5 h and 3 h, respectively (Heldmaier et al. 2024). These URs are present at thermoneutrality as well as during cold exposure and require 22 through 38% of the daily energy budget. We analyzed the effect of torpor on the expression of these URs to see if they are involved in metabolic depression during torpor, and if they play a role in timing of torpor.

URs are commonly generated by cellular metabolism. They are synchronized between cells and several brain areas have been identified to generate URs of hormones and activity (for review see Goh et al. 2019). Despite the widespread occurrence of URs in cellular metabolism, endocrine and neuronal activities, it is not known if they are in involved in systemic control of metabolic rate, and if they are also involved in metabolic depression during torpor.

The sympathetic and parasympathetic nervous systems plays an important role in the peripheral control of blood flow and metabolism, assuming a major role for torpor induction, maintenance and arousal. A parasympathetic influence on torpor induction and maintenance was concluded from heart rate irregularities but the significance of these findings for signalling the entire depression of physiological functions during torpor are still debated (Morhardt 1970, Lyman and O’Brien 1988, Milsom et al 1999, Zosky 2002). Arousal from torpor is clearly associated with high sympathetic activity and nonshivering thermogenesis by brown adipose tissue that is activated by ß-adrenergic stimulation with noradrenaline (Heldmaier 1969; Feist 1970; Osborne et al. 2005).

Mice lacking noradrenaline (dopamine ß-hydroxylase knockout) do not enter torpor (Swoap et al 2006). This inability could be restored when mice were treated with a ß3-specific adrenergic agonist, assuming that sympathetic adrenergic signalling is also required for torpor induction. This is supported by the suppression of torpor in Djungarian hamsters that received a single injection of 6-hydroxydopamine, which transiently inactivates sympathetic innervation (Braulke and Heldmaier 2010). These results suggest a wider role of ß-adrenergic signalling for the induction of torpor behaviour, beyond triggering of arousal (Swoap and Wineshenker 2008). To further elucidate this role we implanted Djungarian hamsters with 10 day release pellets of Mirabegron, a ß3-specific adrenergic agonist, and another group received 10 day release pellets of Propranolol, a blocker of ß-adrenergic receptors.

## Methods

### Animals

Djungarian Hamsters (*Phodopus sungorus*) were bred and raised as described previously (Heldmaier et al. 2024). They received food (Ssniff V2140-000) and water ad libitum. At the age of three months they were kept singly in standard Makrolon cages, Type 3, and transferred from long photoperiod (L:D 16:8 h) and 23 °C T_a_ to short photoperiod (L:D 8:16 h) at 23 °C T_a_. Body mass and fur colour index were recorded at weekly intervals to follow acclimation to short photoperiod. Hamsters were checked two times per week during morning hours for the occurrence of torpor by visual inspection or by measuring surface temperature from outside the cage with IR thermography (PeakTech 5610, Ahrensburg, Germany). Following 2 months in short photoperiod first torpor episodes were observed.

Hamsters that repeatedly showed torpor bouts were transferred to a climate chamber at short photoperiod and 15 °C. They were kept in ventilated IVC cages (Zoonlab, Castrop-Rauxel, Germany, type 1 long (volume 10 L)), with little bedding material (wood shavings 80 g) and two paper towels which they gnawed to build a nest. Cages were cleaned and bedding material renewed at weekly intervals. The transfer to 15 °C and IVC cages interrupted torpor behaviour for a few days. When torpor behaviour continued the hamsters were implanted with transmitters for T_b_ and locomotor activity (*n* = 16).

### Implantation of transmitters for T_b_ and locomotor activity

Transmitters for simultaneous measurement of T_b_ and activity (Vitalview 4000; Starr Life Sciences, Oakmont, PA, USA) were implanted with the same procedure as described previously for other abdominal transmitters (e.g. Braulke et al. 2008, Heldmaier et al. 2024). Hamsters were anaesthetized with Rompun (1 mg/kg) and Ketanest (50 mg/kg), and during surgery anaesthesia was controlled and maintained with Fluothane. The transmitter was implanted into the abdominal cavity without fixation. Typically, the hamsters aroused from anaesthesia within 20 min, walked around, and started drinking and feeding. They were returned to their cages and were left for 5 days for full recovery before the recording of MR, Tb and locomotor activity was started.

### Experimental procedure and implantation of drug pellets

The first 12…15 days of recording were used as control period before hormone treatment. Hamsters then received hormone pellets (Innovative Research of America, 12 day release pellets, pellet diameter 2.25 mm) releasing the ß-blocker Propranolol (*n* = 8, 3.6 mg/pellet) or the ß3-agonist Mirabegron (*n* = 8, 0.7 mg/pellet). The pellets were implanted with a trochar s.c. in the neck region of the hamsters. For implantation they were shortly anaesthetized with Fluothane (initial 4% then lowered to 1%), a small skin incision was made between the shoulder blades and the pellet was inserted 2 cm caudally from the incision. The incision was closed with 1 or two knots of suture.

The hamsters were returned to their cages, recovered quickly and the response to hormone treatment was measured for a period of 12 days. Recordings continued and the following 15 days were considered as control period. This procedure of internal and individual control before and after treatment was chosen to cope with the high individual variability of torpor behaviour.

### Metabolic rate

Metabolic rate (MR, mL O_2_ min^−1^) was measured with CaloBoxes (Phenosys GmbH, Berlin, Germany) that were directly connected with the lid of ventilated cages (Zoonlab, Castrop-Rauxel, IVC, type 1 long) (Elfers et al. 2022). Sample air was drawn from the animal cage with flow rates of ~ 85 L/h. Each cage was directly connected with one CaloBox to obtain a high resolution of MR recordings. The CaloBox measures O_2_-, CO_2_-, and water vapor content every 4 s and calculates consumption of O_2_, production of CO_2_, water vapour, respiratory exchange ratio (RER) and heat production (HP). HP was calculated by using the equation HP[mW] = (4.44 + 1.43* RER)* MR[mLO_2_ h^−1^] which provides the oxidative energy retrieved from mixed combustion of carbohydrates and lipids (Heldmaier 1975). Zero adjustment with reference air occurred every 15 min. Results were collected and stored every 30 or 60 s (further details see Heldmaier et al. 2024).

### Body temperature and activity

T_b_ and activity from implanted transmitters were recorded every minute. In one cage we additionally used a thermovision-camera (Optris PI450, thermal resolution 0.04 K, Optris GmbH, Berlin) to locate the hamster and to measure activity and temperature once per sec. This was required for the observation of its behaviour at the beginning of torpor. The thermal image of the camera was split into three observation areas. Area 1 was tracing the hamster and evaluated its maximum T_sf_, which ranged between 20 and 36 °C depending upon the posture of the euthermic hamster. The latter values were close to core temperature when the eyes and nostrils of the hamster pointed towards the camera lens. Area 2 and 3 monitored the cage bottom outside the nest. Their maximum T_sf_ was ~ 15 °C for most of the time because chamber T_a_ was controlled at 15 °C. When the hamster was active outside the nest the T_sf_ in area 2 or 3 increased and matched hamster maximum T_sf_. Activity was further recorded when the hamster was out of sight for the camera and T_sf_ (area 1) dropped to ~ 15 °C. This happened when the hamster climbed the food tray or gnawed the air inlet tubing behind the water bottle. Total activity was obtained from the IR image by calculating the standard deviation (SD) of hamster T_sf_ per min as a running SD in steps of 1 s along the entire data set of 84,600 values per day. For further details see Heldmaier et al. 2024.

During torpor hamsters retreated into their nests. The small amount of bedding material allowed to observe hamster movements inside the nest and to measure T_sf_ while entering torpor.

### Ultradian rhythms

Long-term simultaneous records of T_b_, activity, and metabolic rate were used to identify ultradian rhythms. To identify regular structures in ultradian rhythms, we performed a Wavelet analysis and calculated the power spectrum (package “WaveletComp” Roesch and Schmidbauer 2018) in R 4.2.2 R Core Team 2022)) of individual 24 h records of metabolic rate, T_b_ and locomotor activity. We mainly preferred wavelet analysis because traditional methods will not be very useful in measuring the true period(s) of ultradian activity patterns (Leise 2013). Further details see Heldmaier et al. 2024.

## Results

### Metabolic rate and ultradian rhythms

**Fig. 1:**
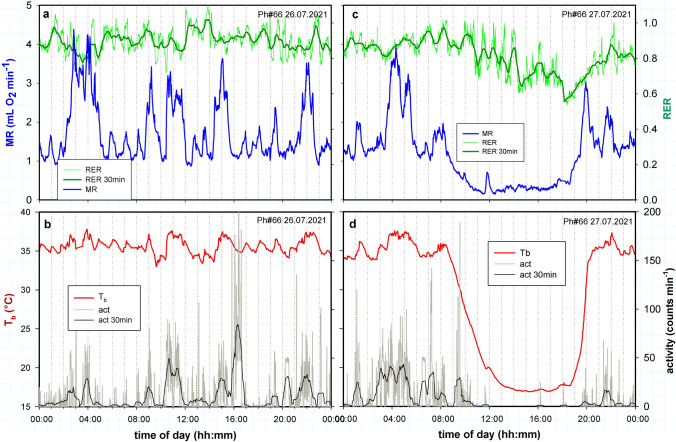
MR, RER, T_b_ and activity of a Djungarian hamster (♀) on two consecutive days at 15 °C T_a_. Day 1 without torpor (**a**, **b**), day 2 with spontaneous torpor lasting ~ 10 h (**c**, **d**). All data were recorded in 1 min intervals. Graph a and b show records of MR (blue) and RER (green) plus 30 min running averages (dark green). Graphs c and d show T_b_ (red) and activity (grey) plus a 30 min running average of activity (black). Body mass was 34.9 g

**Fig. 2:**
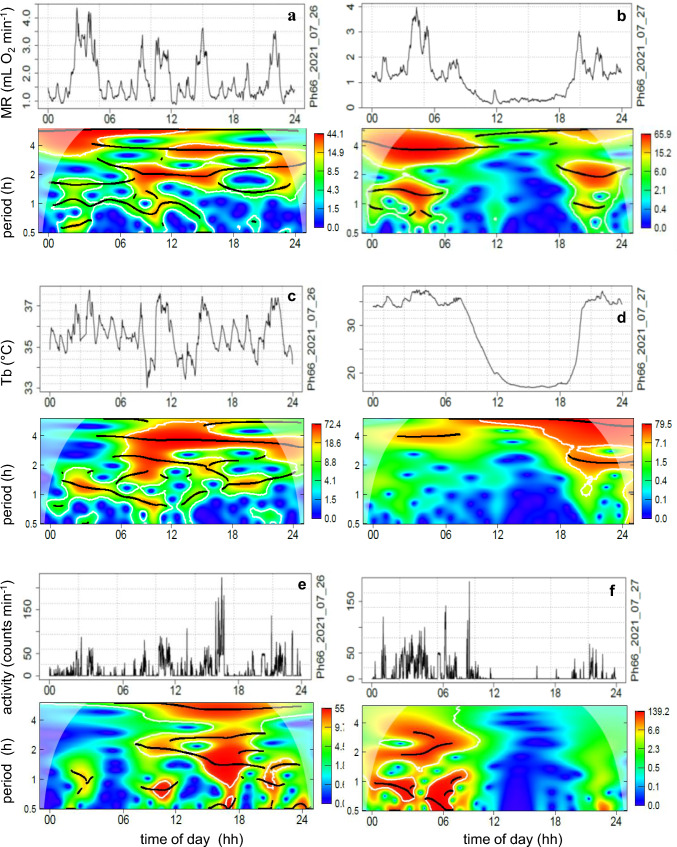
Wavelet analysis of 24hourly records of MR, T_b_ and activity on two consecutive days (**a** + **b**; **c** + **d**; **e** + **f**) from the hamster presented in Fig. 1. Each graph shows in its top section the raw data of 24hourly recordings of MR, T_b_ or activity and in the section below a heatmap of the corresponding wavelet analysis. Red/orange colours in the heatmap indicate significant ultradian rhythmicity. Periods (left y-axis) and wavelet power levels (right y-axis). Ridges of ultradian periods are shown as black lines

The awake state is characterized by ultradian variations of MR, T_b_ and activity (Fig. 1a, b). Spontaneous entry into torpor caused a major reduction of MR by about 70% below the level of RMR and a decrease of T_b_ from 35 to 16 °C (Fig. 1c, d). The decrease of MR was accompanied by an attenuation or loss of ultradian rhythms (URs) during entrance into torpor. The low level of MR and T_b,_ paralleled with the absence of metabolic URs, are the most obvious markers for the presence of torpor. During torpor URs with periods < than 5 h were abolished in all three physiological parameters, in MR as well as T_b_ and activity (Fig. 2). The T_b_ of this hamster lacked short period URs already before entering torpor. It displayed only one UR with a 4 h period (UR*large*), which was interrupted during torpor. The torpid state was terminated by an arousal, i.e. a rapid increase of MR, T_b_ and activity (Fig. 1b, d) and a restart of URs.

During torpor the RER gradually decreased to 0.7 during torpor and arousal, indicating a fuel shift from carbohydrates to lipids. However, this substrate shift took place with some delay. The RER remained high during the initial phase of entrance into torpor (Fig. 1c, green line) but decreased during maintenance of the torpid state and remained low during arousal. It gradually returned to the initial level after completion of arousal. This corresponds with earlier reports of RER in torpid hamsters (Heldmaier et al 1999; Diedrich et al. 2023). A delayed decrease of the RER suggests that during entrance into torpor all metabolic pathways were inhibited at first instance, which was gradually replaced by a shift to lipid combustion during continued torpor.

Entrance into torpor was accompanied by an attenuation of ultradian bursts of metabolic rate visible in the original record (Fig. 1). This could be confirmed by wavelet analysis of the MR record of this hamster (Fig. 2a, b). The heatmaps further reveal that URs of T_b_ (Fig. 2c, d) as well as activity (Fig. 2e, f) are attenuated or disappear during torpor, in parallel with URs of MR.

**Fig. 3:**
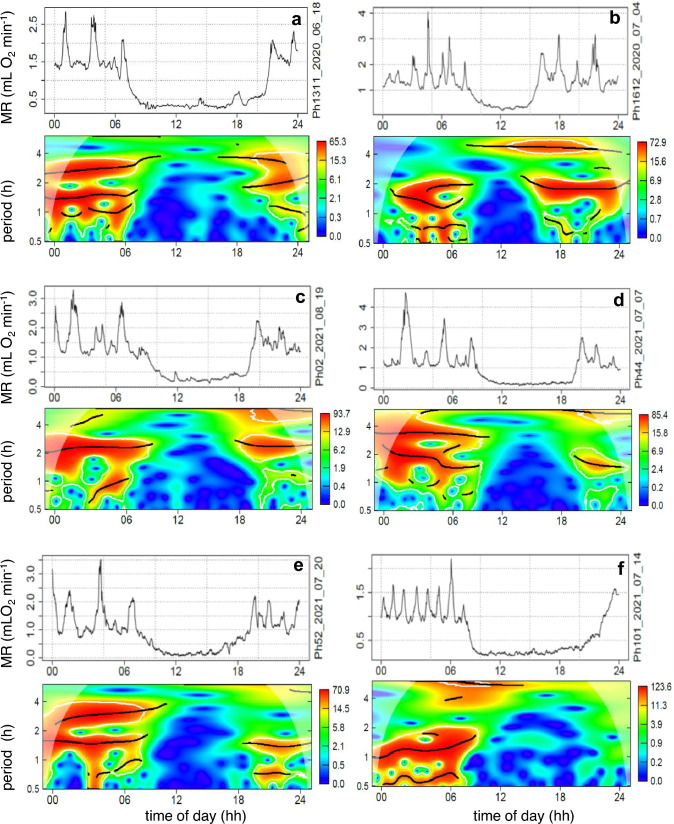
Wavelet analysis of 24hourly records of MR during torpor. Six examples of MR depression during torpor in six Djungarian hamsters are shown (**a** ♂, **b** ♀, **c** ♂, **d** ♂, **e** ♂, **f** ♂). Each graph shows in its top section the raw data of MR and in the heatmap below the corresponding wavelet analysis of the 24hourly record. Red/orange colours indicate significant ultradian rhythmicity. Ridges of rhythms are shown as black lines

An attenuation or disappearance of URs was found in all torpor bouts. This is demonstrated by metabolic URs in further 6 torpor bouts from 6 different hamsters (Fig. 3). The heatmaps in Figs. 2 and 3 show that metabolic URs were maintained up the timepoint of torpor entrance. During torpor entrance short period URs ranging between 0.5 and 2.5 h (UR*small* and UR*medium,* see Heldmaier et al. 2024) disappeared entirely. URs with longer periods disappeared with delay or even continued during torpor. Changes of MR during torpor (e.g. Figure 3e, c) were not detected as UR by wavelet analysis. The heat maps further show a sudden reappearance of URs during arousal. In one example torpor lasted almost until midnight and the reappearance of URs was not completed prior to midnight (Fig. 3f).

### Details of entrance into torpor

**Fig. 4:**
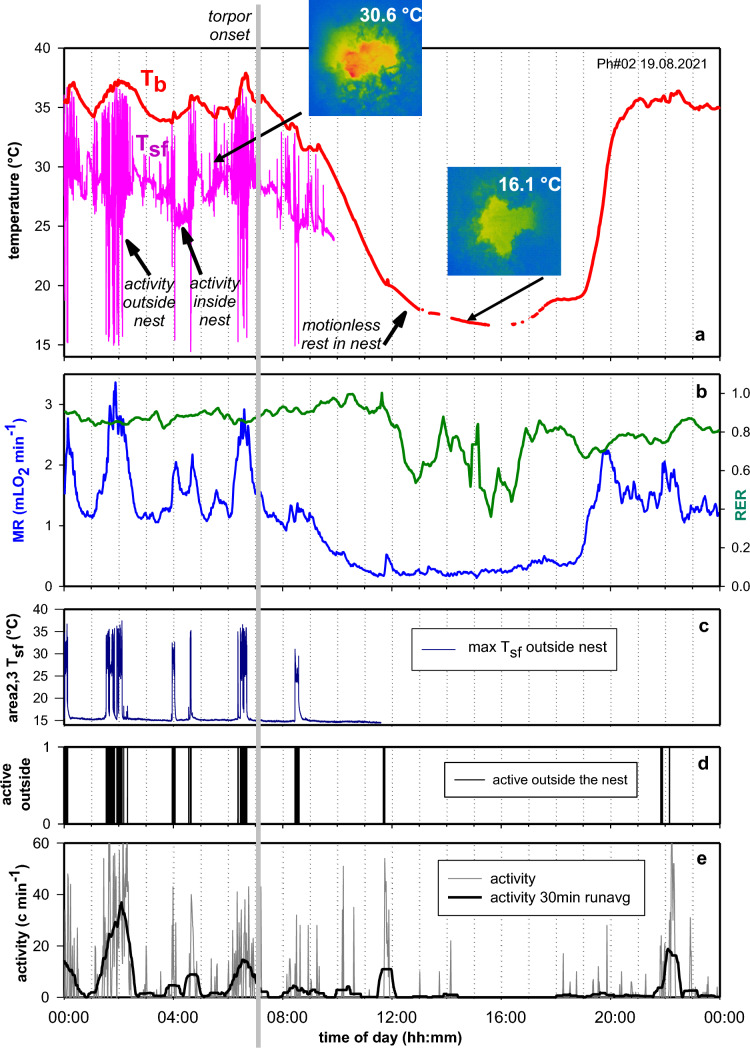
Body core temperature (T_b_, °C min^−1^) and surface temperature (T_sf_, °C sec^−1^) development during torpor (Graph a). The small inserts show IR pictures of the hamster while active inside the nest and while torpid in the nest. Maximum hamster T_sf_ matched hamster core T_b_ when he exposed eyes and face to the thermovision camera. Graph b: Metabolic rate (MR, raw data, mLO_2_ min^−1^) and respiratory exchange rate (RER, 30 min running average). Graph c: T_sf_ of cage bottom outside the nest including hamster T_sf_ when active outside the nest. Graph d: activity outside the nest calculated from T_sf_. Graph e: activity recorded from i.p. transmitter (c min^−1^). The grey vertical bar across all graphs marks the beginning of behavioural quiescence (see T_sf_ graph a) which was assumed as the starting point of torpor

The immediate physiological and behavioural reactions during entrance into spontaneous torpor are largely unknown because handling the animal or any kind of disturbance will almost certainly interrupt the process. This information can only be obtained with telemetry and contact-free methods like IR thermography (Fig. 4). T_sf_ of the hamster is a sensitive indicator for body movements. IR thermography can “see” hamsters inside the nest (see inserts in Fig. 4a) and changes of maximum T_sf_ per sec indicate body movements. Large fluctuations of T_sf_ < 10 °C indicate that the hamster was active outside the nest, moderate amplitude fluctuations > 2 °C were associated with activity inside the nest, small fluctuations of 0.5 °C indicate that the hamster rested motionless in his nest because this amplitude of T_sf_ fluctuations is typical for the cage bottom (Fig. 4c). Motionless periods of rest occurred frequently during torpor for periods of up to an hour. Outside torpor motionless periods were rarely observed and lasted only for a few minutes. Occasionally, T_sf_ approached T_b_ recorded from implanted transmitters when eyes and nostrils of the hamster were in sight of the camera. The IR image further allowed location the hamster in the cage, which was used to determine times for activity in- or outside the nest (Fig. 4c, d). Activity outside the nest was accompanied by an increase in MR, T_b_ and major peaks of activity. The 24hourly activity pattern retrieved from T_sf_ and the thermal image closely paralleled the ultradian locomotor activity pattern recorded with implanted transmitters (Fig. 4e).

Entrance into torpor is a transient process which takes longer than arousal from torpor. Mechanisms involved in triggering the onset of a torpor bout are so far unknown. It may be helpful to identify any physiological or behavioural responses that characterize the beginning of a torpor bout. The hamster shown in Fig. 4 started torpor with behavioural quiescence (07:15 to 07:55) in its nest. Simultaneously MR and T_b_ decreased below resting MR and T_b_ observed during euthermia before. This suggests that behavioural quiescence is associated with onset of torpor and its beginning was labelled with a grey bar (Fig. 4). The further entrance into torpor was interrupted several times by bursts of activity and MR and the hamster even left its nest for a few minutes while T_b_ was < 20 °C.

**Fig. 5:**
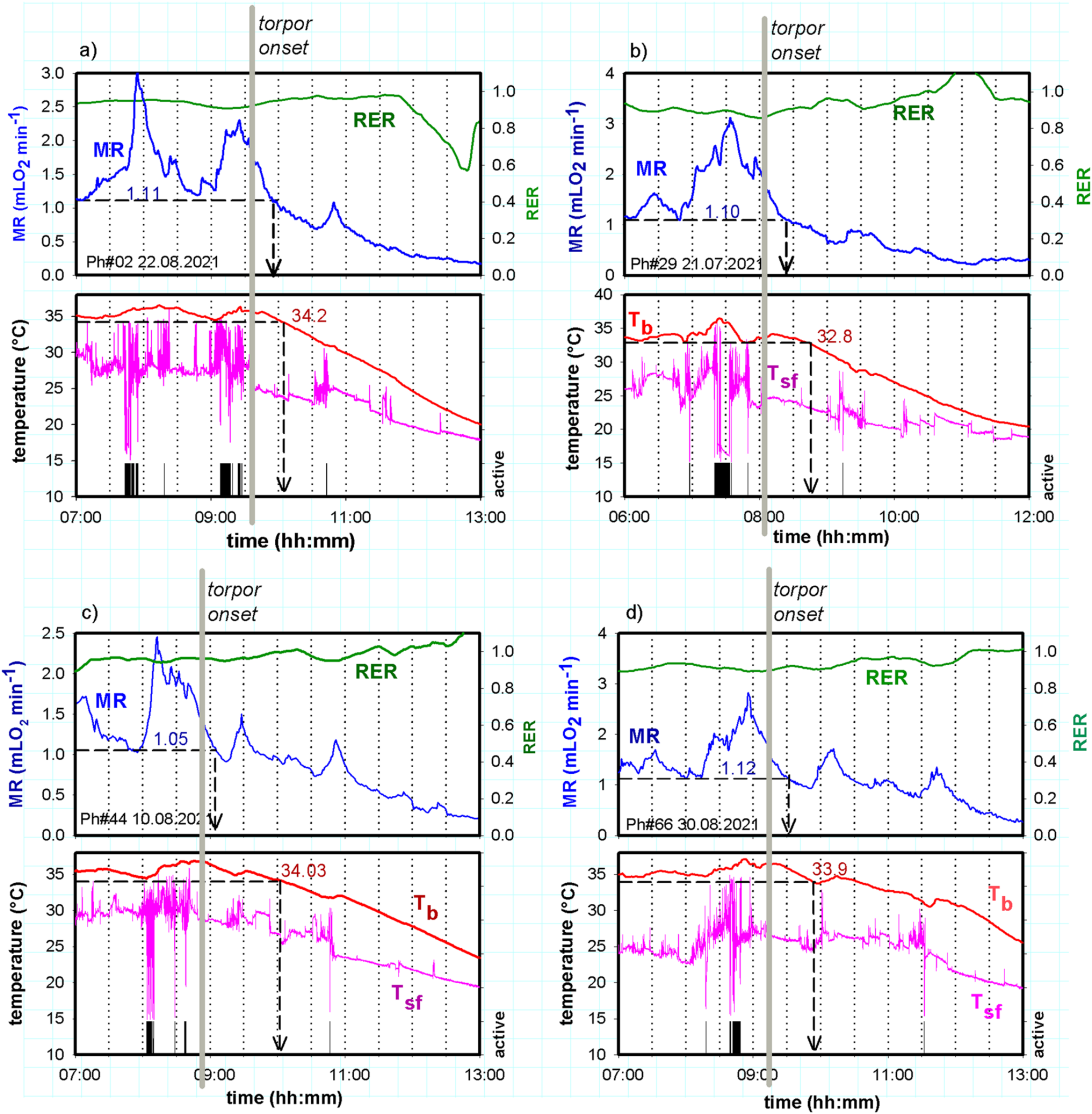
Four examples for torpor entrance in Djungarian hamsters (graph **a**, **b**, **c**, **d**). Each panel consists out of two sections. The upper sections show MR and RER during early entrance into torpor, and the lower section corresponding values of hamster T_b_, T_sf_, and activity outside the cage. T_sf_ variations > 2 °C indicate movements, variations < 0.5° indicate motionless quiescence of the hamster. Vertical grey lines mark the beginning of behavioural quiescence and postulated torpor onset. Dashed horizontal lines in the upper sections of graph **a**, **b**, **c**, **d** show minimum MR (= RMR) during the euthermic phase (00:00….00:07 h). Dashed vertical arrows mark the time when current MR decreased below euthermic minimum MR. Dashed horizontal lines in the lower sections of graphs **a**, **b**, **c**. **d** show minimum T_b_ during the euthermic phase (00:00….00:07 h). Dashed vertical arrows mark the timepoint when T_b_ went below the euthermic minimum T_b_

The relevance of behavioural quiescence for torpor onset is elucidated when comparing it with metabolic and thermal degression during early entrance into torpor (Fig. 5). Each of the four examples includes two sections, an upper section with MR and RER, and a lower section for T_b_, T_sf_ and activity. All four hamsters were active outside the nest and had a peak MR before entering torpor. While MR returned to RMR the hamsters entered an extended period of motionless rest > 15 min which was marked as onset of torpor (grey bar). During this period of behavioural quiescence MR and T_b_ started or continued to decline below euthermic RMR and T_b_ of the hamster. The dashed arrows in the upper sections mark the time when current MR decreased below this level. The dashed vertical arrows in the lower sections mark the time when current T_b_ decreased below its euthermic threshold level. MR passed the threshold of MR 17.5 ± 4.0 min after the beginning of behavioural quiescence. Decreasing T_b_ passed the threshold 44.7 ± 17.2 min after the beginning of behavioural quiescence.

Combined recordings of T_sf_, MR, RER, T_b_ and activity were obtained from 4 hamsters on a total of 65 days. They showed full torpor bouts on 28 days and attempts to enter torpor on 5 days, i.e. a 50.8% incidence torpor behaviour during the observation period of 65 days. An initial period of behavioural quiescence following a burst of MR could be identified in 24 torpor bouts (85.7%). In 4 torpor bouts this initial phase could not be clearly identified (14.3%). Properties of the 24 torpor bouts are summarized in Table 1. The torpor bouts lasted 10.05 h, and the hamsters reached a minimum MR of 0.161 mLO_2_ min^−1^, which was 15% of minimum MR during activity and 8.9% of mean MR during the activity phase. The initial period of behavioural quiescence occurred 39.4 min after an MR peak and lasted 24.8 min. This concludes that torpor induction preferably includes a combination of at least five processes (1) peak MR prior to torpor entrance, (2) behavioural quiescence, (3) depression of MR and related physiological and biochemical processes, (4) decrease of T_b_, and (5) attenuation of ultradian rhythms as shown in the chapters before.

**Table 1:** Properties of torpor bouts and timing of entrance into torpor after peak MR

	Mean ± SD
MR peak before entering torpor (mL O_2_ min^−1^)	2.83 ± 0.35
MR minimum during active phase (mL O_2_ min^−1^)	1.074 ± 0.049
MR mean during active phase (mL O_2_ min^−1^)	1.809 ± 0.161
MR minimum during torpor (mL O_2_ min^−1^)	0.161 ± 0.062
T_b_ minimum during active phase (°C)	34.04 ± 0.37
T_b_ min during torpor (°C)	17.99 ± 0.98
Torpor bout duration (min)	603 ± 65
Timing of torpor onset following MR peak (min)	29.4 ± 6.5
Duration of behavioural quiescence (min)	24.8 ± 3.8

Mean values from 24 (*n* = 24) torpor bouts of 4 hamsters (*n* = 4). Individual means were calculated for each hamster and these individual means were used to calculate an overall mean ± SD

14.3% of torpor bouts started without an extended period of behavioural quiescence after an ultradian MR peak. We deliberately defined a minimum duration of 15 min to separate it from other short periods of behavioural quiescence during the active phase of hamsters. It may be that shorter periods of behavioural quiescence may be sufficient or that this step is substituted by other processes to induce entrance into torpor.

### Effect of propranolol and mirabegron

**Fig. 6:**
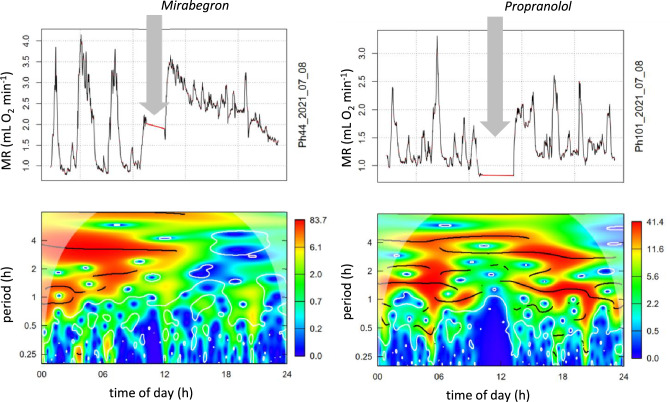
Acute metabolic and thermal responses of hamsters to subcutaneous implantation of ß-adrenergic agents. Graph **a** and **b**: Implantation of the ß3 agonist Mirabegron. Graph **c** and **d**: Implantation of the ß-blocker Propranolol. Graphs a and c records of MR. Records were interrupted while hamsters were removed from their cage for implantation (grey arrows). Graphs b and d: period duration of URs from MR in graphs a and c. Red/orange colours indicate significant ultradian rhythmicity. Ridges of rhythms are shown as black lines

Hamsters received subcutaneous implants with Propranolol (ß-adrenergic antagonist) or Mirabegron (ß3-adrenergic agonist) to analyse the role of the ß-adrenergic system on ultradian rhythms and torpor control. Mirabegron immediately increased MR, reduced the amplitude of URs and decreased RER, indicating lipid combustion for nonshivering thermogenesis via ß3-adrenergic receptors of brown adipose tissue (Fig. 6, Table 2). This was a transient effect which disappeared within 24 h.

**Table 2:** Short term responses to Mirabegron and Propranolol

	Mirabegron			Propranolol		
	Before	After	*p* (*n*)	Before	After	*p* (*n*)
MR [mLO_2_ min^−1^]	1.723 ± 0.078	2.584 ± 0.22	*p* < 0.002 (7)	1.394 ± 0.171	1.527 ± 0.175	*n.s.* (5)
HP [mW]	570.1 ± 29.8	844.1 ± 71.0	*p* < 0.001 (7)	478.6 ± 58.9	520.6 ± 62.0	*n.s.* (5)
T_b_ [°C]	35.71 ± 0.2	35.80 ± 0.35	*n.s.* (8)	35.61 ± 0.16	36.06 ± 0.25	*n.s.* (7)
RER	0.918 ± 0.019	0.705 ± 0.008	*p* < 0.001 (7)	0.903 ± 0.027	0.863 ± 0.026	*n.s.* (5)
Activity [counts min^−1^]	7.28 ± 0.64	5.39 ± 0.78	*p* < 0.032 (8)	7.583 ± 1.103	7.148 ± 0.909	*n.s.* (7)

Mean values of MR, RER, *T*_b_ and activity were calculated for the hours from midnight to the implantation of Mirabegron or Propranolol (= before) and the hours after the implantation until midnight (after). Statistical analysis was performed by ANOVA for repeated measures and in case of significance the Holm–Sidak method for individual responses was applied (Sigmastat 3.5)

**Fig. 7:**
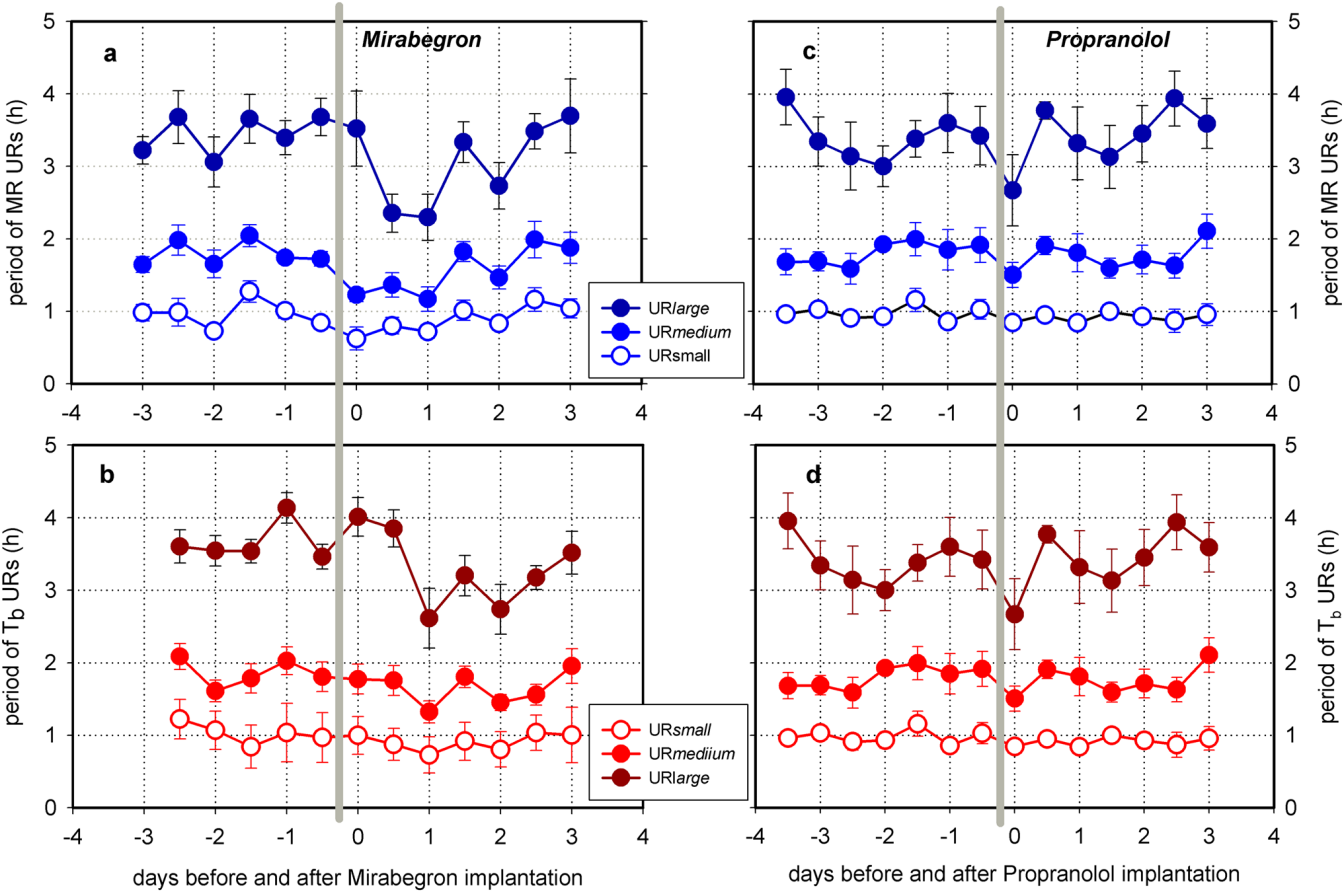
Effect of Mirabegron (**a**, **b**) or Propranolol (**c**, **d**) on ultradian rhythms of MR and T_b_ in Djungarian hamsters. Mean values ± SEM, Mirabegron n = 6, Propranolol *n* = 7. Wavelet analysis was performed on 12 h sections through three days before and 3 days after drug implantation. Mirabegron treatment caused a transient shortening of the large ultradian rhythms of MR (**a**) and T_b_ (**b**) for three days. The difference between the day before and the day (**e**) after the implantation was significant (paired *t*-test, *n* = 6, *p* < 0.01). Mirabegron also reduced the amplitude of large ultradian rhythms (**f**) by 65% (paired *t*-test, *n* = 6, *p* < 0.001)

Wavelet analysis revealed that UR*large* and UR*medium* for MR as well as T_b_ had shorter periods under the influence of Mirabegron (Table 3). This was also a transient response which disappeared within 3 days. UR*small* was not affected by Mirabegron (Fig. 7a, b). Propranolol had no effect on the UR periods in MR and T_b_ (Fig. 7c, d). Only a small deflection was observed on the day of implantation, which may have been caused by handling, anaesthesia and surgery.

**Table 3:** Mirabegron effects on URs of MR and T_b_

	UR period before (h)	UR period with Mirabegron (h)	
MR UR*large*	3.495 ± 0.462	2.716 ± 0.410	*P* = 0.031 (*n* = 6)
MR UR*medium*	1.790 ± 0.178	1.417 ± 0.169	*P* = 0.009 (*n* = 6)
MR UR*small*	0.973 ± 0.203	0.853 ± 0.179	*n.s.* (*n* = 6)
T_b_ UR*large*	3.867 ± 0.325	2.998 ± 0.620	*P* = 0.011 (*n* = 7)
T_b_ UR*medium*	1.826 ± 0.143	1.573 ± 0.217	*P* = 0.037 (*n* = 7)
T_b_ UR*small*	0.953 ± 0.158	0.839 ± 0.115	*n.s.* (*n* = 7)

Mean periods were calculated for two days before and two days after Mirabegron implantation. Values are means ± SD. Significance was tested with paired *t*-test

**Fig. 8:**
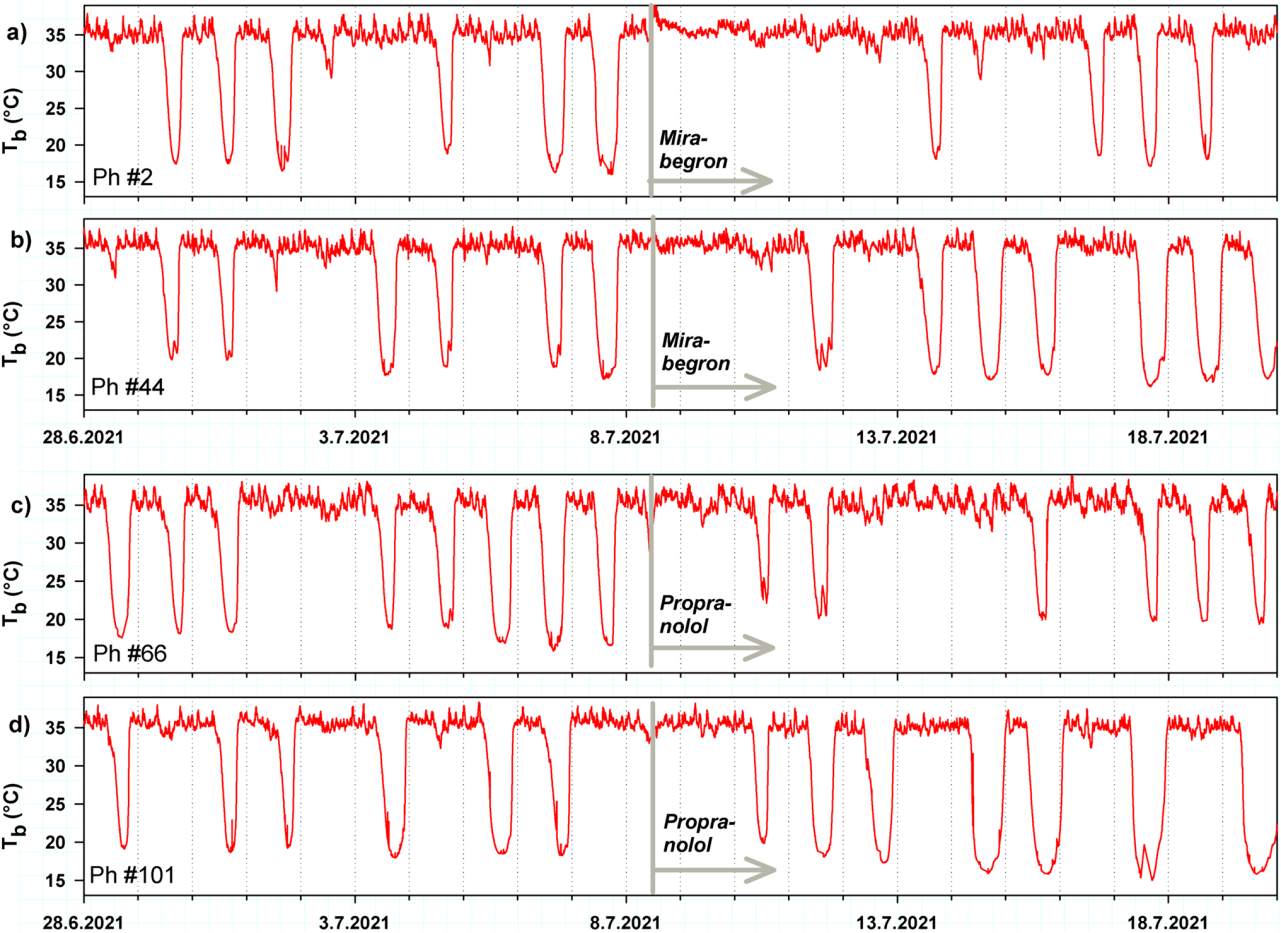
Long term effects of Mirabegron and Propranolol on torpor behaviour of Djungarian hamsters. Time course of T_b_ recorded in 2 hamsters 10 days before and 10 days after implantation of Mirabegron (**a**, **b**) and 2 hamsters implanted with Propranolol (**c**, **d**)

Examples of 20 day records of torpor behaviour in four individual hamsters before and after hormone implantation confirm the transient modulation of URs following Mirabegron implantation (Fig. 8a, b). The apparent delay of torpor restart following Mirabegron implantation, as compared to torpor restart following Propranolol, could not be confirmed statistically because two hamsters in each treatment group failed to show torpor during the 10 day observation period following implantation.

## Discussion

Torpor is characterized by profound changes in the entire physiology of an animal. Hibernators and animals practicing daily torpor can spend a large amount of their lifetime in this condition, 10 h per day during daily torpor or up to 11 months with multi-day torpor bouts during hibernation (Hoelzl et al 2015). Two major states of vigilance are currently differentiated in animals, sleep and wakefulness. They can be differentiated by major changes in behaviour and physiological regulation. The behavioural and physiological adjustments during torpor do not match with either of these two states of vigilance, proposing that torpor should be considered as a separate, third state of vigilance, allowing animals to bypass unfavourable periods and periods with shortage of food or water supply.

### Torpor entrance and metabolic depression

During torpor entrance most hamsters showed a specific sequence of events. Following a peak of locomotor and metabolic activity they settled quietly in their nest. During this period of behavioural quiescence MR and T_b_ decreased below minimum MR- and T_b_-levels in euthermia (Fig. 5). This sequence was observed in 85.7% of 28 torpor bouts, whereas in 14.3% torpor was entered without an extended period of rest following peak MR. Metabolic depression was accompanied by a suppression of URs. During the initial period of metabolic depression, the RER remained constant at a high level around 0.94 suggesting that during entry into torpor, at first all metabolic processes were slowing down. When torpor continued the RER gradually decreased towards 0.7, suggesting a shift from carbohydrate to lipid combustion, which was maintained through arousal. This is in accordance with previous observations (Heldmaier et al. 1999; Diedrich et al. 2023).

Diedrich et al. (2023) showed that blood glucose levels decreased after the beginning of a torpor bout, reached lowest values of 80 mg% after about 3 h, and returned to normal values during arousal. Reductions of blood glucose level were also observed in fasting induced torpor in mice (Lo Martire et al 2018), as well as during spontaneous torpor in hibernating species like hedgehogs (Al Badry et al. 1983, Hoo-Paris et al. 1982), squirrels (Galster et al. 1975), hamsters (Weitten et al. 2013) and bats (Heldmaier et al. 1969). The decrease in blood glucose level in the torpid state could be interpreted as a sign of substrate shortage, which facilitates or induces torpor. However, the delayed reduction of RER and blood glucose levels during entrance into torpor alternatively suggests that these reductions were not the cause of torpor but were actively controlled as part of metabolic pathway adjustment during torpor.

### Ultradian rhythms

Ultradian cycling of MR, T_b_ and activity disappeared during entrance into torpor. All three URs of MR were attenuated or completely abolished. URs with short periods of 0.5 through 2.5 h (UR*small*, UR*medium*) disappeared immediately, whereas URs with periods > 5 h disappeared slowly or even continued through torpor. All URs restarted with arousal from torpor and returned to a similar pattern as prior to entrance into torpor. During the course of torpor occasional rises of MR, T_b_ and activity were observed which, however, never reached the euthermic level of MR and T_b_. These interruptions were not detected as regular events by wavelet analysis, neither for itself nor linked to pre- or post-torpor URs. This may be due to a temperature effect on ultradian rhythms, or a dependency upon the general level of metabolic rate, similar to the effects of temperature on the expression of human circadian rhythms (Malan and Heldmaier 2023). At present it remains an open question whether torpor affects only the peripheral expression of URs or if ultradian oscillators in cells and tissues are suppressed during torpor.

Multi-day torpor bouts during hibernation of ground squirrels in a thermally constant environment still showed circadian T_b_ rhythms with a low amplitude (Grahn et al. 1994; Ruby et al. 2002). The timing of entrance and arousal in hibernating European hamsters correlated with circadian rhythms (Malan et al. 2010), indicating that the circadian oscillator is maintained during torpor. Therefore it is not unlikely that repeated bursts of MR, T_b_ and activity during torpor are based on the continued activity of ultradian oscillators. At first sight these occasional disturbances of torpor appear contradictory because they will waste energy, but they may be important for the induction of spontaneous arousal from daily torpor.

Our present findings shed new light on the nature of torpor induction and metabolic depression. Entrance into torpor is not simply a shut-down of metabolic heat production, but it also inhibits ultradian cycling of metabolism. The combination of both measures reduces metabolic heat production and facilitates lowering of T_b_. It is not known how depression of MR is controlled but the different time course of RMR and URs suggests that two different neuronal control systems are involved. This adds to the complex systemic reorganisation of body functions during torpor including behavioural adjustments, suppression of ultradian rhythms, reduction of MR, blood pressure, ventilation, reorganisation of metabolic pathways towards lipid combustion, inhibition of translation, transcription and protein degradation (Andrews 2019; van Breukelen and Martin 2002; Berriel Diaz et al. 2004; Heldmaier et al. 2004, Squire et al. 2003; Martin et al. 1999), reduction of mitochondrial ATP-production (Staples et al. 2022), and the shift of T_b_ control to lower levels of temperature (Heller et al. 1977). The EEG becomes silent early during entrance into daily torpor as well as during multiday torpor bouts in hibernation (DeBoer and Tobler 1994; Heller 1979).

### Targeted induction of torpor

Torpor is a powerful measure to save energy for survival in unfavourable environments, suggesting that shortage of food or energy reserves may serve as a proximate or ultimate signal for torpor induction. Laboratory mice are the only species known where torpor can occur as an immediate response to food removal (Dikic et al. 2008; Oelkrug et al. 2011). Other species respond with delay by the prolongation of torpor bouts or an increasing torpor incidence (Ruf et al. 1993; Lovegrove et al. 2001; Giroud et al. 2008). Several attempts have been made to induce torpor by the injection of metabolic inhibitors, to find out if any specific shortage of substrate or the inhibition of metabolic pathways could induce torpor behaviour. These included peripheral application of 2-Deoxy-D-glucose to inhibit glycolysis or mercaptoacetate to inhibit fatty acid utilisation (Dark and Miller 1997; Dark et al. 1999), or general metabolic inhibitors like H_2_S (Blackstone et al. 2005, Jensen et al. 2021) or the nucleotide AMP (Swoap et al. 2007). These treatments caused transient metabolic depressions of animals but not to the extent as it is known from natural torpor. Torpor-like responses could also be obtained by manipulations of endocrines related to energy balance, e.g. the central application of Neuropeptide Y (Paul et al. 2005) or injections with 3-Iodothyronamine (Scanlan 2004; Braulke et al. 2008). All treatments may have scratched some facet of torpor metabolism but failed to induce sustained torpor behaviour (Bouma et al. 2012).

The sympathetic nervous system plays a central role in control of heart rate, blood pressure, tissue blood flow, cellular metabolism and ventilation, i.e. body functions which provide the basis for metabolism and heat control of the body. Mice with a dopamine β-hydroxylase knockout, which lack the ability to produce the SNS transmitters noradrenaline and adrenaline failed to enter starvation induced torpor. Treatment with a synthetic β3 agonist restored their ability to enter torpor (Swoap et al. 2006). 6-Hydroxydopamine transiently inhibits hormone release from sympathetic nerve endings. In Djungarian hamsters, 6-Hydroxydopamine treatment inhibited torpor behaviour for about a week (Braulke and Heldmaier 2010). This supports the necessity of β-adrenergic signalling for the expression of torpor behaviour. In the present study we tried to confirm this with subcutaneous implants of propranolol, a blocker of β-adrenergic signal transmission. In contrast to our expectations, the treatment with propranolol had no effect on torpor behaviour nor on the amplitude and frequency of URs, except for the hours immediately following subcutaneous implantation which could be an artefact of handling and anaesthesia. This negative result could be a dosage or pellet-release problem and requires further studies.

In a further attempt we tried to enhance torpor behaviour by treating the hamsters with the β3-specific agonist mirabegron. It enhanced MR probably because ß3-adrenergic stimulation elicited nonshivering thermogenesis in brown adipose tissue. It also lowers the RER to 0.7 indicating that metabolic pathways were rerouted rapidly from glucose to lipid utilisation. Some responses to Mirabegron suggest that it can modulate URs, e.g. a reduction of amplitude and period of URs. However, this transient effect disappeared after a few days and was weaker than required for long term control of torpor behaviour.

None of the metabolic inhibitors, nor hormonal treatments, nor manipulations of ß-adrenergic signalling as in our present study, induced a response which compares to natural torpor (Bouma et al. 2012). This suggests that either the chemical master-switch for torpor has been missed so far, or that torpor initiation requires the interaction of several neuronal networks and endocrine activities. The latter is more likely since torpor is based on numerous adjustments of cellular, systemic and behavioural processes.

### Torpor, sleep and CNS control

Daily torpor occurs during the resting phase of the circadian cycle. This timing is obvious in the present study but was also found in different species of mice and marsupials (Godfrey 1966; Hill 1975; Hut et al. 2011; Oelkrug et al. 2011; Grimpo et al. 2013). Sleep preferably occurs during the resting phase of the circadian cyle, suggesting a functional relationship between sleep and torpor. EEG patterns during entrance into torpor in mice were found “indistinguishable from NREM sleep” (Huang et al. 2021). This indicates a similarity between sleep only during the initial phase of torpor entrance while during continued torpor at low T_b_ the EEG cannot be detected anymore (Deboer and Tobler 1995). Arousal is followed by an extensive period of slow-wave sleep (Trachsel et al. 1991; Strijkstra and Daan 1997; Deboer and Tobler 1994, 2003, Palchykova et al. 2002, Vyazovskiy et al. 2017) while URs were restarted with high amplitudes like during the active phase of the circadian cycle. This concludes that sleep and torpor are different physiological processes but their similarity during entrance into torpor may need further exploration.

The silencing of URs during torpor entrance suggests a link with the CNS network responsible for UR generation. In vivo experiments with hypothalamic ablation and in-vitro studies suggest that URs can be generated by cells and tissue slices and that these cellular responses are coordinated within tissues (Isomura 2014; Yang et al 2022). There is also evidence for coordinating networks (Grant et al. 2018) as well as a central origin and control of ultradian rhythmicity in the PVN (Gerkema et al. 1990; Wu et al. 2018, Heldmaier et al. 2024). EEG studies in rats revealed URs in rat brain electrical activity which coincided with release rates of neurotransmitters in several brain areas as measured with push–pull superfusions in rats and cats. The central application of agonists and antagonists of catecholamine receptors and histamine receptors prolonged or shortened the period of URs, and α_1_-antagonists even abolished EEG URs in the posterior hypothalamus of the rat (Philippu 2019). The same result was achieved by electrocoagulation of the rostral arcuate nucleus (Grass et al. 1996). This underlines the significance of a central origin and control of URs. A further exploration of CNS control of metabolic URs is required for a better understanding of metabolic depression torpor.

## Abbreviations

DUO: Dopaminergic ultradian oscillator
MR: Metabolic rate (oxygen consumption [mLO_2_ min^−^^1^])
RMR: Resting metabolic rate
RER: Respiratory exchange ratio
MR: Metabolic rate (mLO_2_ min^−^^1^)
PVN: Paraventricular nucleus
QRFP: Pyroglutamylated RFamide peptide
SPZ: Subparaventricular zone
T_b_: Body temperature (abdominal)
T_sf_: Surface temperature
TSH: Thyroid stimulating hormone
TRH: Thyroid releasing hormone
UR: Ultradian rhythm
UR*small*: Ultradian rhythm with small amplitude
UR*medium*: Ultradian rhythm with medium amplitude
UR*large*: Ultradian rhythm with large amplitude
